# Pooled RNA-extraction-free testing of saliva for the detection of SARS-CoV-2

**DOI:** 10.1038/s41598-023-34662-2

**Published:** 2023-05-08

**Authors:** Orchid M. Allicock, Devyn Yolda-Carr, John A. Todd, Anne L. Wyllie

**Affiliations:** 1 Department of Epidemiology of Microbial Diseases, Yale School of Public Health, New Haven, CT 06510 USA; 2 SalivaDirect, Inc, New Haven, CT 06510 USA

**Keywords:** Viral infection, Laboratory techniques and procedures

## Abstract

The key to limiting SARS-CoV-2 spread is to identify virus-infected individuals (both symptomatic and asymptomatic) and isolate them from the general population. Hence, routine weekly testing for SARS-CoV-2 in all asymptomatic (capturing both infected and non-infected) individuals is considered critical in situations where a large number of individuals co-congregate such as schools, prisons, aged care facilities and industrial workplaces. Such testing is hampered by operational issues such as cost, test availability, access to healthcare workers and throughput. We developed the SalivaDirect RT-qPCR assay to increase access to SARS-CoV-2 testing via a low-cost, streamlined protocol using self-collected saliva. To expand the single sample testing protocol, we explored multiple extraction-free pooled saliva testing workflows prior to testing with the SalivaDirect RT-qPCR assay. A pool size of five, with or without heat inactivation at 65 °C for 15 min prior to testing resulted in a positive agreement of 98% and 89%, respectively, and an increased Ct value shift of 1.37 and 1.99 as compared to individual testing of the positive clinical saliva specimens. Applying this shift in Ct value to 316 individual, sequentially collected, SARS-CoV-2 positive saliva specimen results reported from six clinical laboratories using the original SalivaDirect assay, 100% of the samples would have been detected (Ct value < 45) had they been tested in the 1:5 pool strategy. The availability of multiple pooled testing workflows for laboratories can increase test turnaround time, permitting results in a more actionable time frame while minimizing testing costs and changes to laboratory operational flow.

## Introduction

During the emergence and spread of the SARS-CoV-2 virus in 2020, the majority of testing for the virus was aimed at diagnosing COVID-19 (the disease that it causes) in patients presenting with symptoms characteristic of COVID-19. However, it was soon recognized that while an infected person could develop COVID-19 disease symptoms 3–8 days post infection, some individuals would never develop symptoms (asymptomatic)^1–3^. Despite their lack of symptoms, asymptomatic individuals can be infectious, carrying viral loads high enough to spread the virus to uninfected individuals. Thus, it became clear that to control the spread of SARS-CoV-2 infection, a two-pronged approach should be used in the general population to prevent virus spread from infected asymptomatic individuals to the non-infected population.

The first prong involved utilisation of physical barriers (e.g. face masks) to minimize virus spread via aerosols^4,5^. The second prong involved routine weekly testing for SARS-CoV-2 in all asymptomatic (capturing both infected and non-infected) individuals at high-risk for infection^6,7^. Such testing was considered critical in situations where a large number of individuals co-congregate such as schools, prisons, aged care facilities and industrial workplaces. Testing strategies relied on obtaining a respiratory tract specimen and an assay for the presence of SARS-CoV-2 antigen or genome.

Molecular tests for the detection of the SARS-CoV-2 virus genome have been generally more sensitive than antigen tests; however, molecular tests can be costly, can take days to result during outbreaks, and can be hard to scale for large population testing. Furthermore, for tests requiring a swab-based respiratory tract specimen, these can be uncomfortable and difficult to obtain, especially in the setting of weekly self-collected specimen protocols, deterring individuals from participating in testing^8^. Early in the pandemic response, saliva emerged as an alternative specimen for SARS-CoV-2 testing and by 2021 it became apparent that a self-collected specimen could obviate the disadvantages of respiratory tract swab specimens. Critically, the clinical sensitivities for SARS-CoV-2 detection were found to be similar between respiratory tract swab and saliva specimens^9^, with saliva more sensitive in the early infection period^10,11^.

To address some of the early limitations of testing, SalivaDirect was developed as an open-source protocol wherein clinical laboratories could adopt a streamlined, easy-to-use, inexpensive, scalable and flexible genomic (RT-qPCR) assay method for SARS-CoV-2 detection^12^. Importantly, the assay was based upon a simple self or observed saliva collection protocol. The SalivaDirect assay was developed to simplify testing individual saliva specimens; however, with the momentum around testing large-scale asymptomatic populations (e.g. school students, faculty and staff), where SARS-CoV-2 prevalence was low, a more scalable protocol was required for sustainable testing programs. We therefore investigated higher throughput protocols, wherein saliva specimens are pooled prior to testing with RT-qPCR. These pooled testing approaches were evaluated for the clinical sensitivity of SARS-CoV-2 detection.

## Results

### Pooling sizes and workflow selection

We initially performed a limit of detection range finding study to determine the impact of sample dilution via pooling using the SalivaDirect RT-qPCR assay on detection sensitivities. As pool size increased the resulting assay cycle threshold (Ct) values increased as well, generally in a linear manner (see Supplementary Fig. S1). The smallest change in Ct values (i.e. loss of assay sensitivity) of pooled versus neat saliva was obtained with 1:5 pooling (1 positive and 4 negative saliva samples). Thus a 1:5 pooling strategy was employed for workflow analysis. Our preliminary results indicated that the SalivaDirect assay could detect SARS-CoV-2 in pooled saliva specimens with high virus loads, but additional testing was required to optimize saliva pooling and processing workflows.

**Figure 1:**
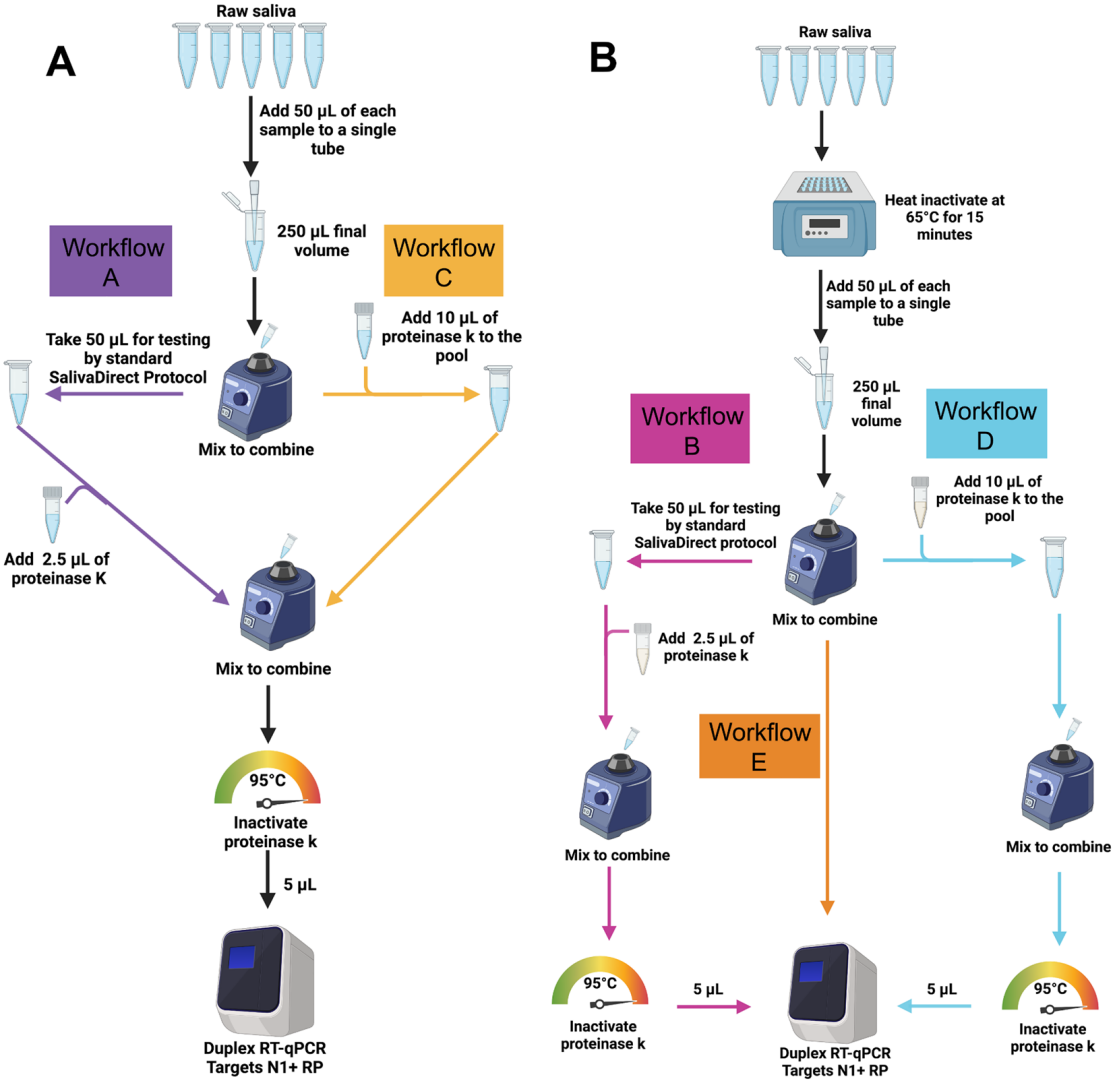
The SalivaDirect pooled testing workflows evaluated in the study. (**A**) For workflows A (purple) and C (gold), 50 µl of SARS-CoV-2-positive saliva and 200 µL total volume of four SARS-CoV-2-negative saliva samples (50 µl each) were pooled and vigorously vortexed to mix. For workflow A, 50 µl of the pooled sample was tested following the standard SalivaDirect protocol^12^. For workflow C the remaining sample was treated with 10 µl of proteinase K then heat inactivated before testing directly without further treatment in the SalivaDirect RT-qPCR assay. (**B**) For workflows B, D and E, individual, non-pooled samples were incubated at 65 °C for 15 min before combining 50 µl of each sample into pools of 5 pre-treated samples. For workflow B (pink), 50 µl of the pool of pre-treated samples was tested through the standard SalivaDirect protocol. For workflow E (orange), 10 µl of each of these pre-treated pools was removed and tested with the SalivaDirect RT-qPCR assay without proteinase k treatment. Finally, for workflow D (blue), 10 µl of proteinase k was added to the remaining volume of the pre-treated pool then heat inactivated before testing in the SalivaDirect RT-qPCR assay.

Extrapolating from previous work^13^, we selected 5 workflows representing different pooling strategies (Fig. 1). Initial analyses using 5 pools showed that 4 workflows (A-D) provided similar results for most of the pools (see Table S1). Workflow E provided a much larger shift in Ct values for all five pools (5.26) and hence loss of assay sensitivity. As expected, a shift in Ct values (to higher) was noted for all five pooled workflows compared the standard SalivaDirect RT-qPCR assay performed on the undiluted positive sample. For workflows A-E, initial analysis of the differences in Ct values between the pools and individual positive samples resulted in a Ct shift of 2.17 to 3.50. Workflow E was omitted from further evaluations.

### Workflow analysis

Twenty-five SARS-CoV-2-positive saliva specimens were used for pooling, with each pool including one unique positive specimen and 4 SARS-CoV-2-negative specimens, to make 25 contrived pools. The Ct values (when initially tested on day of saliva collection) for the SARS-CoV-2-positive samples ranged from 22.98 to 39.43. The majority (40–45%) of the specimens had Ct values > 35 indicating a relatively low concentration of virus, 28–32% of the specimens had Ct values < 30 indicating a higher concentration of virus (see Table 1, Supplementary Table S2).

**Table 1:** Distribution of the Ct values of clinical saliva samples used for pooling.

Ct range*	No. samples workflow A (n = 22)	No. samples workflows B–E (n = 25)
20.0–29.9	7 (32%)	7 (28%)
30.0–34.9	5 (23%)	8 (32%)
35.0–40.0**	10 (45%)	10 (40%)

*Samples < 40 Ct are considered positive for SARS-CoV-2.

**Mean Ct for the CFX96 Touch RT-qPCR instrument when determining LOD for analytical sensitivity using this set of reagents was 36.7.

**Figure 2:**
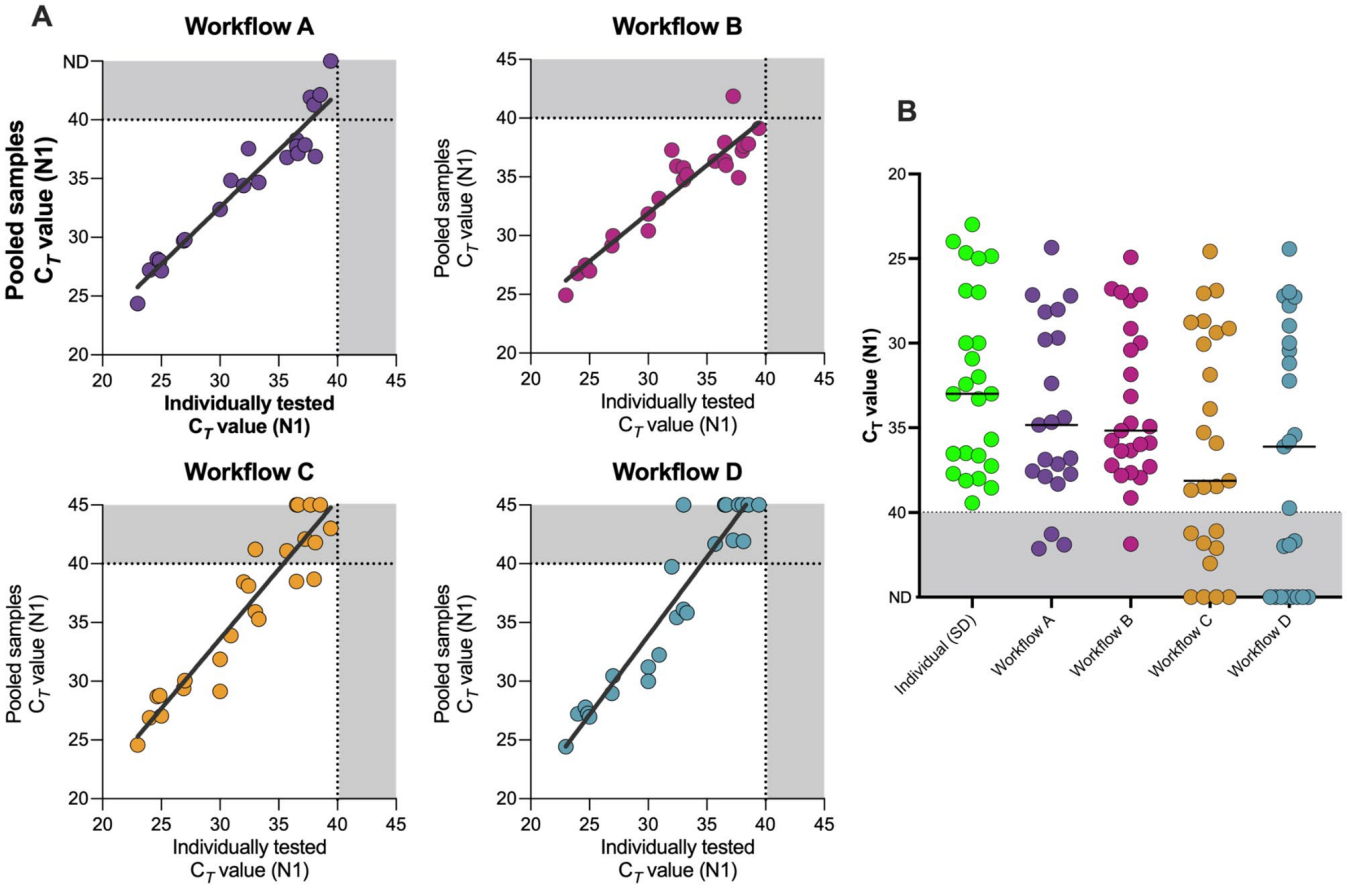
SARS-CoV-2 N1 gene detection with individual saliva-based RNA-extraction-free RT-qPCR testing versus pooled sample testing using Workflows A–D. (**A**) N1 detection of one positive sample pooled and tested with equal volumes of 4 negative samples correlated to the Ct value obtained when samples were tested individually. (**B**). Ct values obtained from each sample tested individually and when combined with 4 negative samples and tested with each of the workflows. For each workflow, the black horizontal line represents the median Ct value of the samples tested. The grey shaded area denotes the region of 40–45 Ct. The N1 Ct cut-off for classifying individual samples as positive is 40 and the PCR is run for a total of 45 cycles. Due to the specific detection of N1, no cut-off was set for the pooled samples.

We assessed the sensitivity of each workflow through the smallest shift in Ct values between the undiluted sample processed with SalivaDirect and the workflow in question, along with the smallest number of pools passing the assay cut-off for positivity (Ct = 40). When compared to undiluted samples processed with the standard SalivaDirect assay, Workflows A and B provided the highest sensitivity (Fig. 2), with Ct values crossing the 40 Ct threshold in 3 and 1 pool(s), respectively. In contrast, workflows C and D demonstrated a lower clinical sensitivity, with SARS-CoV-2 detection lost in 8/25 and 10/25 pools processed by these workflows, respectively.

Overall, workflow A resulted in an average positive agreement of 88.6% (86.4% and 91.0% for the individual test replicates), compared to single sample testing results using the standard SalivaDirect protocol. Workflow B resulted in a 98% positive agreement (100% and 96% for the individual test replicates), compared to single sample testing (Fig. 2A). The positive agreement for workflows C and D was lower, with averages of 76% and 62%, respectively.

A theoretical Ct shift of Log_2_(n) can be estimated for most RT-qPCR tests due to the dilution of positive samples when pooled with negative samples. For pools of 5, a Ct value shift of 2.3 would be expected. The Ct shift observed for Workflows A and B were below this expected value, with Ct value shifts of 1.99 and 1.37 respectively, confirming a slight loss of assay sensitivity. Workflows C demonstrated the largest Ct value shift of 2.81.

### Impact of pooling on clinical sensitivity

**Figure 3:**
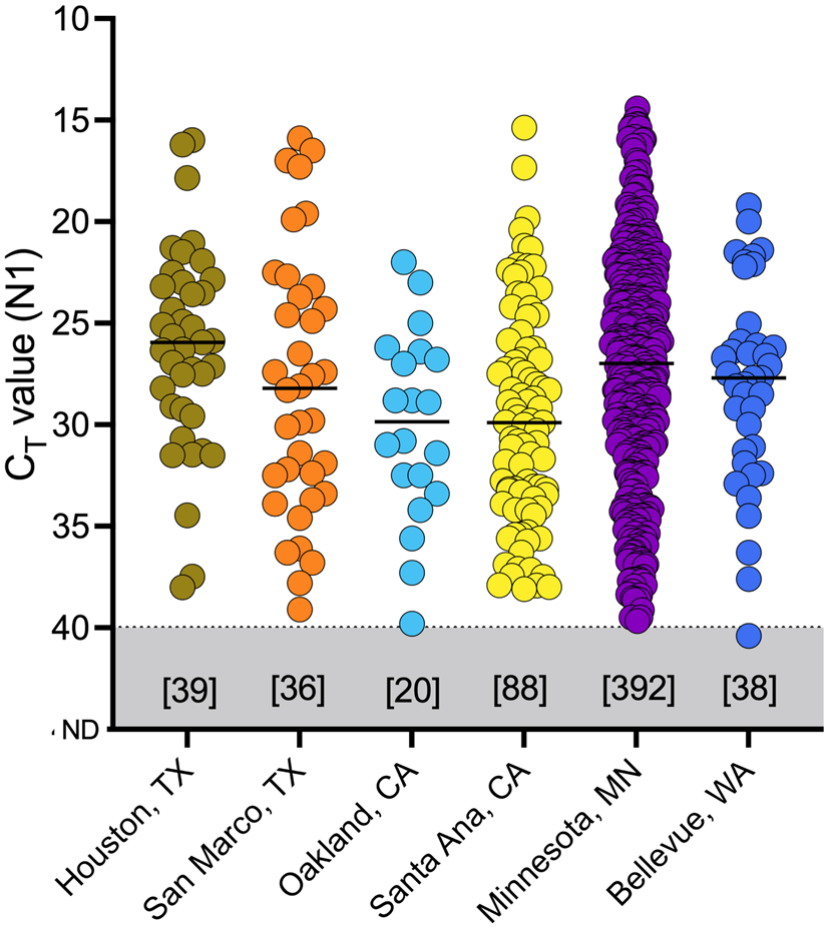
Detection of SARS-CoV-2 N1 gene persists when Ct value shift from workflow A is applied to data from six clinical laboratories across the US in July 2021. Each dot represents the clinical samples. The black horizontal lines indicate the median Ct value of the samples from each laboratory. The number of positive samples reported from each site is reported in the square brackets above the location of the laboratory.

To determine the pragmatic loss of clinical sensitivity with pooling before performing the SalivaDirect RT-qPCR assay with workflows A-D, we queried six SalivaDirect CLIA laboratories across the United States for the Ct values obtained from sequential testing of saliva samples for SARS-CoV-2. These values and the breakdown of samples per site are presented in Fig. 3 (see Supplementary Table S3). The average Ct value from all results provided by the six labs was 28.0. There was no statistical difference in the Ct values reported across the six labs. Out of a total of 613 positive samples, only 16 samples (2.6%) had Ct values between 38 and 40—a rate similar to findings reported from India when the sensitivity of pooled saliva testing was also assessed^14^. Considering a worst-case Ct shift in pooling workflows A and B of 1.99 (see Workflow Analysis results, above), and if all of the 613 reported samples had been tested using either of workflow A or B, the 16 weakly positive samples would have shifted in Ct to between 40 and 42. While the parameters recommended for individual samples testing would have classified these samples as negative, due to the specificity of N1 detection and the results from the validation study indicating the possibility for virus detection in this range, for pooled testing we recommend reflex testing of any pool generating a Ct value < 45.

## Discussion

The widespread surveillance of asymptomatic individuals proved instrumental for controlling the spread of SARS-CoV-2. Pooling of samples before testing provides a resource-saving approach to increase testing capacity, especially for surveillance in a population with a low infection rate^13,15^, such as travellers, school populations and employees of large organisations. Sample pooling also serves communities where the general availability of tests remains limited or cost-prohibitive to the general populace^16^. Even if only testing a certain fraction of the population, these members of the community can serve as a proxy to the broader community, perhaps identifying larger outbreaks through family members and their associated activities. As saliva is easy to collect from a large number of people, pooling strategies are thus a natural extension to surveillance programs^17^. While pooling saliva does impact assay sensitivity and potentially decrease virus detection, as also observed by others^14,18–20^, we found that the actual impact appears to be minimal, likely due to the dilution of possible contaminants in certain saliva samples that can have an inhibitory effect on the PCR reaction^21^.

However, despite the resource-saving benefits^14,15,18,22^, clinical laboratories have been hesitant to implement pooled sample testing^23^ due to: (1) stringent workflows which do not fit within existing laboratory operations, (2) a lack of clear guidance on how to implement such methods and (3) the perception that clinical sensitivity of the assay will be lost with pooling. The methods we propose in the current study are straightforward extensions of a simple SARS-CoV-2 testing method and can be easily conducted manually, without requiring additional investment. SalivaDirect is a flexible extraction-free platform for RT-qPCR testing. For ease of implementation and safety of lab personnel, multiple workflows^24^ were developed for the testing of individual samples. We sought to extend this level of flexibility for labs seeking to offer pooled testing and found that workflows A and B provided the best sensitivity of SARS-CoV-2 detection. As such, workflows A and B were selected as the proposed approaches for pooling of the SalivaDirect test, with B providing a heat pre-treatment step for labs who require it by local Environmental Health and Safety guidelines. Moreover, the initial heat pre-treatment step of workflow B improves the viscosity of the saliva samples, meaning that not only is the sample easier to pipette, but when vortexed prior to pooling, this creates a more homogenous sample, thus improving the chance of virus detection^25–27^. When followed by the second heating step (the inactivation of proteinase K), the two heat treatment steps combined further mitigate the expected effect of sample dilution^28^ through the degradation of potential PCR inhibitors present.

Since weakly positive samples (Ct values 38–40) may be missed when testing pools of larger sizes (pools > 5), we evaluated six datasets comprising 613 SARS-CoV-2 positive samples from clinical diagnostic laboratories across the U.S with the calculated relative sensitivity loss resulting from pooling applied. Using such real-world data, we found that pooling saliva in groups of 5 samples prior to testing is expected to have minimal impact on clinical sensitivity. Based upon the lab-reported Ct values, application of the SalivaDirect pooling workflows A or B, proposed in the current study, only 2.6% of these positive samples would have shifted across the assay cut-off for individual sample testing, to Ct values 40–42. Importantly, if these samples were pooled, none would have become undetectable (Ct > 45). Based on our observations and recognizing the specificity of the N1 primer–probe assay, we therefore recommend that any sample pool producing a Ct value between 40 and 45 should be retested individually using the standard SalivaDirect protocol.

Surveillance programs for SARS-CoV-2 (and other pathogens of interest) in low prevalence populations must be operationally pragmatic, removing barriers to both implementation and sustainability, particularly in limited-resource settings. First, they need to be cost-effective. Pooled testing significantly reduces reagent costs, lab personnel cost, and lab resource needs. We have previously estimated that the cost of reagents to perform the SalivaDirect assay ranges from ($2–4/sample) using retail non-discounted pricing from the various manufacturers^12^. Thus, the 5 sample pooling approach should reduce the reagent cost to $0.4–0.8/sample. Since reagents are the costliest component of qRT-PCR assays (especially when complete “assay kits” are considered), the SalivaDirect pooled protocols could be of significant benefit to large-scale testing efforts in high- and low-income countries, alike. Second, these programs need to be easy to implement. Self-collection of a simple saliva specimen obviates the need for healthcare workers to collect specimens and the associated personal protective equipment. Third, programs should utilize existing resources for sample collection. Self-collection of saliva can be performed anywhere and the resulting specimen can be deposited at a drop site location (e.g. school or workplace entrance) such that specimens from thousands of participants are collected in a parallel manner. Pooling of specimens once received in the laboratory for testing should fit into established laboratory accessioning and pre-analytic workflows. Finally, the end test results must provide acceptable clinical sensitivities and specificities. We have shown that a saliva-based RNA-extraction-free pooled (1:5) testing strategy results in detection of 97.4–100% SARS-CoV-2-positive samples, as compared to individual sample testing. Large pooled testing programs have successfully demonstrated the efficacy of pooled saliva testing for helping to keep schools safely open^15,29^, with pooled samples having a similar sensitivity to the molecular testing of individual samples, in terms of both qualitative and quantitative (comparable Ct values between pooled and individual samples) measures. It is important to note however, that during times of high virus prevalence, when a greater number of tests are expected to return a positive result, pooled testing strategies must be frequently reviewed and when necessary, updated to minimize the number of reflex tests required which can negate the resource-savings benefits of pooled testing^13,19,22,27^.

Overall, surveillance testing is not generally easy, requiring a pivot by traditionally clinical diagnostic labs, especially when scalable protocols are not commonly used. Thus, when a decrease in positive cases is observed, there is a psychological and practical desire to decrease testing efforts. However, these dips in regards to COVID-19 cases can lead to a decrease in preventative measures, which inevitably leads to disease resurgence. Additionally, with the ongoing emergence of different SARS-CoV-2 variants of concern, the need for affordable and sustainable mass testing strategies remains, and should be considered necessary throughout all world regions^16^. Our findings suggest that combining saliva with a practical pooling protocol facilitates surveillance testing efforts, especially in resource-limited settings^16^. Such pooled testing has the potential to significantly reduce the overall number of tests and associated costs and can likely be more broadly applied to other respiratory pathogens of interest. This would in turn operationally permit an increased frequency of testing meaning an increased likelihood of detecting individuals earlier in their infection, mitigating pathogen transmission. This approach, originally developed to support broader screening in schools and workplaces for SARS-CoV-2, provides a foundation for managing future outbreaks of upper respiratory pathogens.

## Methods

### Ethics statement

The use of de-identified saliva specimens from healthy or asymptomatic individuals was approved by the Institutional Review Board of the Yale Human Research Protection Program (Protocol ID. 2000028394)^30^. Study participants were informed in writing about the purpose and procedure of the study, and consented to study participation through the act of providing the saliva sample; the requirement for written informed consent was waived by the Institutional Review Board. Additionally, the Institutional Review Board of the Yale Human Research Protection Program determined that the use of de-identified, remnant COVID-19-positive clinical samples obtained from laboratory partners for the RT-qPCR testing conducted in this study is not research involving human subjects (Protocol ID: 2000028599). All experiments were performed in accordance with the Institutional Review Board of the Yale Human Research Protection Program guidelines and regulations.

### Sample collection

All de-identified saliva samples used in the current study were collected unsupplemented into simple laboratory plastic tubes per the SalivaDirect protocol^31^. All samples were tested with the SalivaDirect assay^32^ in our research laboratory to confirm SARS-CoV-2 status. Samples were stored at -80 °C until further analysis.

### Pooled sample testing

To understand the effect of sample dilution by pooling on clinical sensitivity, a total of 20 saliva specimens which previously tested positive with the modified CDC assay RT-qPCR assay, with resulting Ct values between 22.98 and 39.43), were diluted 1:5, 1:10, and 1:20 with negative saliva specimens from healthcare workers^13^. Undiluted specimens and pools were tested with the standard SalivaDirect RT-qPCR assay.

After identifying the optimal pool size, we performed an initial workflow evaluation using 5 different pooled samples, each composed of a single SARS-CoV-2-positive saliva sample pooled with 4 SARS-CoV-2-negative saliva samples, in a 1:5 dilution. To confirm our initial workflow findings and assess the sensitivity of viral detection when pooling, 20 additional pools (1 positive with 4 negative saliva specimens) were prepared and tested using the five different workflows.

The five different saliva pooling workflows investigated in both the initial and confirmation studies are depicted in Fig. 1. All saliva samples were thawed on ice prior to testing and all samples were tested in duplicate. For workflows A and C (Fig. 1A), 50 µl of each sample, (including the SARS-CoV-2-positive saliva) were pooled to 250 µL total volume, followed by vigorously vortexing to mix. For workflow A, 50 µl of the pooled sample was tested following the standard SalivaDirect protocol^12^. For workflow C the remaining sample was treated with 10 µl of proteinase K then heat inactivated before testing directly without further treatment in the SalivaDirect RT-qPCR assay. Workflows B, D and E (Fig. 1B) involved incubating individual non-pooled samples at 65 °C for 15 min before combining 50 µl of each sample into the pools of 5 pre-treated samples. For workflow B, 50 µl of the pool of pre-treated samples was tested through the standard SalivaDirect protocol. For workflow E, 10 µl of each of these pre-treated pools was removed and tested with the SalivaDirect RT-qPCR assay without proteinase K treatment. Finally, for workflow D, 10 µl of proteinase K was added to the remaining volume of the pre-treated pool then heat inactivated before testing in the SalivaDirect RT-qPCR assay.

From the resulting Ct values, we first calculated the ΔCt value for each sample, being the shift in Ct value from when tested individually as compared to pooled testing. Subsequently, we calculated an overall ΔCt value for each pooled testing workflow, being the average of the shift in Ct values for all samples tested.

### Assessment of clinical Ct values with pooling

To evaluate the real-world potential loss of sensitivity on clinical samples with pooling, we estimated the average change in Ct value for each pooled testing workflow using the results from the 25 sample workflow validation study. We then estimated how the change in Ct value would potentially impact assay sensitivity by applying the ΔCt value (with pooling) to real-world SalivaDirect RT-qPCR SARS-CoV-2 testing results. Six SARS-CoV-2 testing sites around the U.S., all testing with the standard SalivaDirect protocol, provided sequential testing results (Ct values for positive specimens) during August 2021 and to these values we applied the ΔCt value that we observed from the validation study.

### Statistical analyses

The correlation of Ct values between each workflow and the individual positive samples was assessed using the Pearson correlation coefficient and represented graphically with linear regression. The negative RT-PCR of the target gene was set at the Ct value of 45 for the statistical analysis. All statistical analyses were performed using GraphPad Prism version 9.0 (GraphPad Software, San Diego, CA). For the calculation of percent positive agreement, samples are considered positive at Ct < 45.

## Supplementary Information

Supplementary Figures.

Supplementary Tables.

## Data Availability

Data from this study is available in the supplemental information.
