# Supplementary material for: Pooled RNA-extraction-free testing of saliva for the detection of SARS-CoV-2
Source: Sci Rep. 2023 May 8;13:7426. doi: 10.1038/s41598-023-34662-2 (PMC10165292; doi:10.1038/s41598-023-34662-2)
Supplement: Supplementary file 1 — Supplementary Figures. [file 41598_2023_34662_MOESM1_ESM.pdf]

SUPPLEMENTAL MATERIAL

Pooled RNA-extraction-free testing of saliva for the detection of SARS-CoV-2

Orchid M. Allicock[1*], Devyn Yolda-Carr[1*], John A. Todd[2], Anne L. Wyllie[1#]

[1]Department of Epidemiology of Microbial Diseases, Yale School of Public Health, New Haven, CT 06510, USA; [2]Flambeau Diagnostics, Madison WI 53719, USA.

[*]These authors contributed equally to this article

[#]Correspondence: anne.wyllie@yale.edu

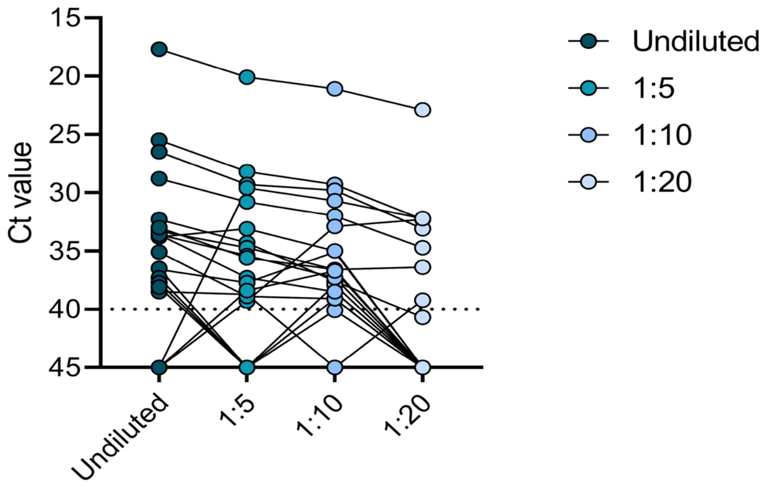

Figure S1. Preliminary findings supporting that SalivaDirect could be used to detect SARS-CoV-2 in pooled saliva specimens. A total of 20 positive saliva specimens were tested with SalivaDirect undiluted, or in pools with 4 (1:5), 9 (1:10), or 19 (1:20) negative saliva specimens. Overall, SARS-CoV-2 could still be detected in pools of up to 20 saliva specimens when initial positive saliva specimens had a Ct value <36. However, some weakly positive saliva specimens were no longer detected when pooled with negative saliva. Potential PCR inhibitors may be present in some saliva specimens as shown by the detection of SARS-CoV-2 in pooled specimens while the undiluted specimen tested negative with SalivaDirect. Shown are the Ct values for the N1 primer-probe set and the dashed line indicates the assay cut-off for determining sample positivity.

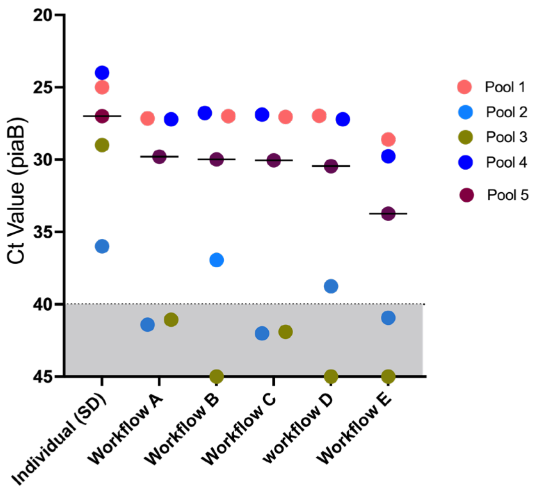

Figure S2. Initial comparison of the Ct values for the individual sample and the pooled samples processed with the 5 alternative workflows. The horizontal dashed line is at Ct = 40, the assay cut-off for individual samples.
